# A study of microbial diversity in a biofertilizer consortium

**DOI:** 10.1371/journal.pone.0286285

**Published:** 2023-08-24

**Authors:** Cristóbal Hernández-Álvarez, Mariana Peimbert, Pedro Rodríguez-Martin, Dora Trejo-Aguilar, Luis D. Alcaraz

**Affiliations:** 1 Laboratorio de Genómica Ambiental, Departamento de Biología Celular, Facultad de Ciencias, Universidad Nacional Autónoma de México, Coyoacán, Mexico City, Mexico; 2 Posgrado en Ciencias Biológicas, Universidad Nacional Autónoma de México, Mexico City, Mexico; 3 Departamento de Ciencias Naturales, Unidad Cuajimalpa, Universidad Autónoma Metropolitana, Mexico City, Mexico; 4 Laboratorio de Organismos Benéficos, Universidad Veracruzana, Veracruz, Mexico; Benemérita Universidad Autónoma de Puebla: Benemerita Universidad Autonoma de Puebla, MEXICO

## Abstract

Biofertilizers supply living microorganisms to help plants grow and keep their health. This study examines the microbiome composition of a commercial biofertilizer known for its plant growth-promoting activity. Using ITS and 16S rRNA gene sequence analyses, we describe the microbial communities of a biofertilizer, with 163 fungal species and 485 bacterial genera found. The biofertilizer contains a variety of microorganisms previously reported to enhance nutrient uptake, phytohormone production, stress tolerance, and pathogen resistance in plants. Plant roots created a microenvironment that boosted bacterial diversity but filtered fungal communities. Notably, preserving the fungal-inoculated substrate proves critical for keeping fungal diversity in the root fraction. We described that bacteria were more diverse in the rhizosphere than in the substrate. In contrast, root-associated fungi were less diverse than the substrate ones. We propose using plant roots as bioreactors to sustain dynamic environments that promote the proliferation of microorganisms with biofertilizer potential. The study suggests that bacteria grow close to plant roots, while root-associated fungi may be a subset of the substrate fungi. These findings show that the composition of the biofertilizer may be influenced by the selection of microorganisms associated with plant roots, which could have implications for the effectiveness of the biofertilizer in promoting plant growth. In conclusion, our study sheds light on the intricate interplay between plant roots and the biofertilizer’s microbial communities. Understanding this relationship can aid in optimizing biofertilizer production and application, contributing to sustainable agricultural practices and improved crop yields.

## Introduction

Food demand has become a crucial concern for humanity’s future due to population growth, resource limitations, and climatic change [1, 2]. The world’s dietary requirements will increase by 62% to 98% by 2050 [2]. Biotechnology benefits food and agricultural production by using innovations to enhance the production process for animals, plants, and microorganisms [2]. Biofertilizers are biotechnological products with microorganisms applied to soil, seeds, or plant surfaces to promote vegetable growth [3, 4]. Optimizing chemical fertilization in crops and transitioning to biofertilizer development is essential due to environmental concerns associated with the excessive use of chemical fertilizers in agriculture. Using chemical fertilizers leads to the accumulation of nitrates in soil and water, which disrupts the nitrogen cycle and contributes to the emission of nitrogen oxides into the atmosphere [5]. Excessive agricultural fertilizers also result in water pollution and greenhouse gas emissions, which can lead to catastrophic events such as the sargassum blooms affecting large areas in the Caribbean [6]. Similarly, excessive using phosphorus fertilizers in agriculture contributes to water pollution, and as phosphorus is an irreplaceable nutrient, it is essential to improve its assimilation in crops [7]. Microbes are capable of phosphorus uptake and assimilation of alternative P sources like phosphonates, and forming mycorrhizal interactions helps to supply a P source to the plant [8, 9]. Favoring microbial consortia, like the ones included in some biofertilizers, affects the carbon cycle, creating a delicate balance between microbial metabolic activity in the plant-microbe interface and biogeochemistry. Such an approach could develop more effective and sustainable biofertilizers [10].

Biofertilizers put microbes to work, particularly in nitrogen fixation and phosphorus solubilization, and help to reduce the need for chemical fertilizers, which could mitigate climate change and improve soil health. Additionally, microbial consortia in biofertilizers could affect the carbon cycle, developing more effective and sustainable biofertilizers [11]. For example, experiments using arbuscular mycorrhizal fungi (AMF) as biofertilizers reduce the application of external fertilizers, phosphorus [12]. In the last decades, using soil microorganisms as biofertilizers have been successful due to their benefits in promoting plant growth, pathogen control, increased quality, and crop yield enhancement [13, 14]. Several companies sell biofertilizers with plant-growth-promoting rhizobacteria (PGPR), *Rhizobium*, and mycorrhizal fungi. However, most commercial preparations advertise their products as general bacterial and fungal compositions (*e*.*g*., Phylum, Class, Order), not declaring a species-level identification [15]. Biofertilizer production traditionally centers on screening, characterizing, and formulating single isolates with the desired plant-growth-promoting traits [16]. Nonetheless, the evidence suggests that bio-inoculants increase their effectiveness when using communities rather than single species [17, 18].

AMF cultivation is challenging as they thrive as symbionts and thus limit *in vitro* cultivation [19]. Trap culture isolates AMF; it uses plants as hosts (traps) growth in soil mixed with sterile sand, usually in pots, and uses plants as baits to attract and host microbes [20–23]. It is also a technique that helps the long-term propagation of AMF [24]. Using plant microbial traps increases microbiome diversity by planting plant hosts into diverse soil samples from the field, then selecting the plant-interacting microorganisms [25]. Since their plant-beneficial effect depends on their ability to colonize roots [26–28], understanding root-microbe interactions is essential for developing new biofertilizers. Trap culture propagation resembles the serial passage across generations in fresh media in experimental evolution. In other contexts, like bioengineering, this is named long-term continuous culture [29]. Experimental evolution involves using known ancestral populations (*e*.*g*., microorganisms) and propagating them over time under selective pressures in managed conditions (*e*.*g*., nutrients, heat). The main goal is to search for the genetic basis of the adaptive traits, resulting in increased fitness under selective conditions [30]. The genetic basis of the adaptations is primarily revealed by high-throughput sequencing [30].

**Fig 1:**
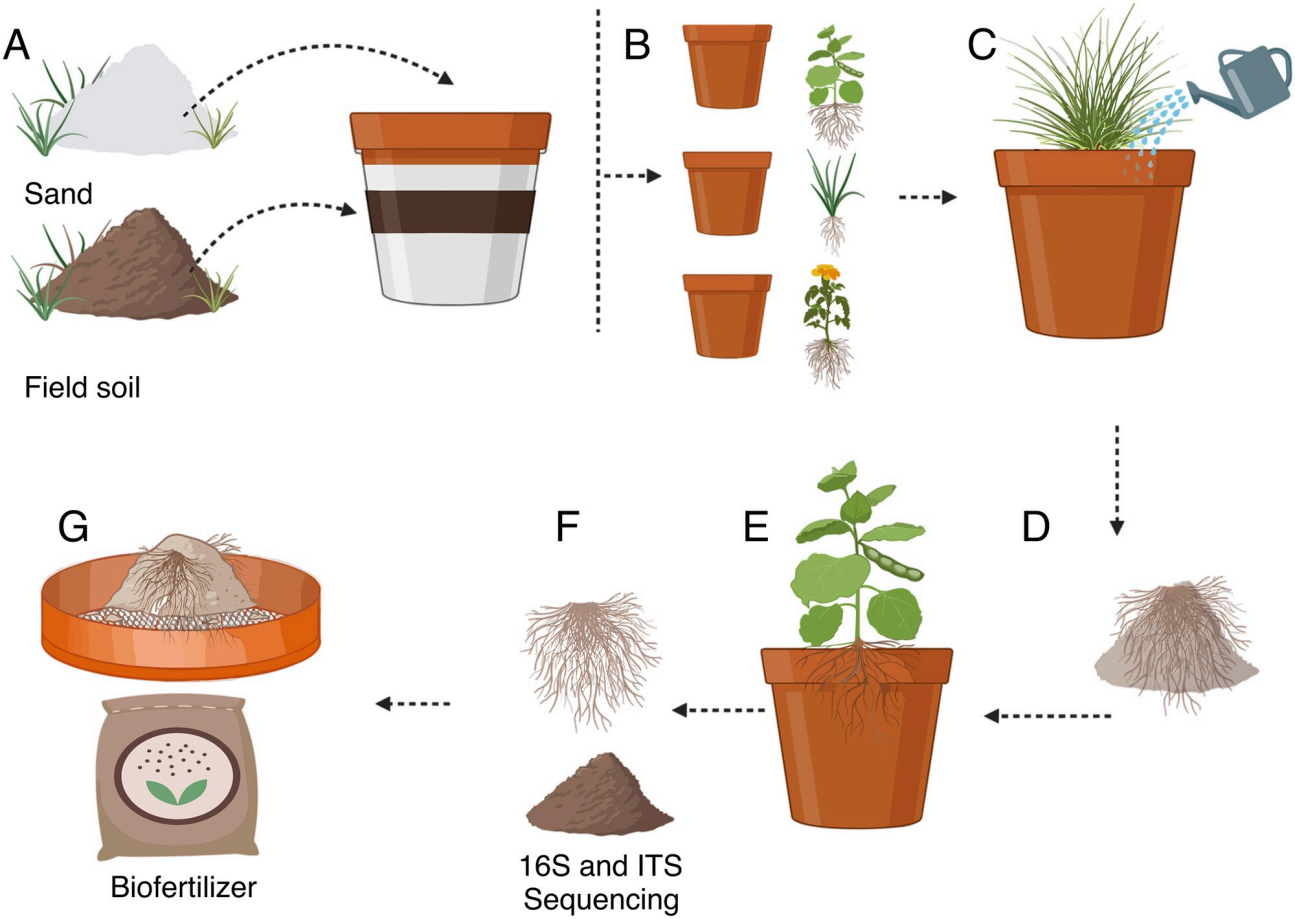
Biofertilizer production overview. Each production cycle spans 16 months. (A) It begins with soil sampling from natural ecosystems (1994), followed by mixing the soil with sterilized sand (three months). (B) Plants are germinated to host arbuscular mycorrhizae and bacteria (three months). (C) The resulting mixture of roots and substrate is then tested for its ability to promote plant growth (four months). (D) Plant-growth promoter consortia are selected and used as inoculum for (E) scale-up production and are kept through plant host rotation to foster microbial diversity. (F) Substrate samples are collected for sequencing analysis before drying, which promotes sporulation. (G) The roots and substrate are sun-dried and ground to produce the biofertilizer (four to five months). This production process helps develop effective biofertilizers with diverse microbial communities that promote sustainable agriculture.

AMF-based biofertilizers are produced through several steps, which include trap cultures from isolates, selection of suitable growth conditions (*e*.*g*., low phosphorus medium), testing, propagating, and scale-up production [19]. Some biofertilizers utilize AMF communities (consortia for engineers) rather than a single AMF species [19]. The development of the biofertilizer described here began as a university spin-off in 1994. Initially, a meticulous collection of 30 soil samples was conducted from diverse natural ecosystems and coffee farms, practicing rustic management. Rustic management involves minimal synthetic inputs, mechanization, and increased labor and traditional methods by coffee farming communities. These coffee farms followed specific practices, including supplying shade, single annual fertilization, manual weeding, abstaining from agrochemical use, and keeping an average production of 1t ha^-1^. Over five years, the soil underwent a processing method developed to generate the biofertilizer, with a detailed protocol published [19]. Selecting the best species consortium was iterated repeatedly (Fig 1). After each cycle, the developer conducted thorough assessments to evaluate the consortium’s plant-growth-promoting efficiency, percentage of mycorrhizal colonization, and the consortium’s mycorrhizal inoculum potential (MIP). Initially, the procedure for obtaining biofertilizers was focused solely on AM fungi (AMF), based only on morphological identification. It was reported that changes from one cycle to another could be shown based on the spores’ appearance [31]. Earlier reports had shown the existence of bacterial and fungal communities living strictly associated with AMF spores, extraradical mycelium, and mycorrhizal roots in the mycorrhizosphere, as well as non-culturable endobacteria inside the spores of some AMF species [32]. Free-living bacteria were also found embedded in the spore wall layers or the micro-niches formed by the peridium hyphae interwoven around the spores in the sporocarps [32]. The constructive interaction between AMF and its microbial communities in the hyphosphere degrades complex organic compounds that AMF cannot degrade by themselves [33].

In this study, we investigated the role of plant roots in selecting microorganism communities responsible for promoting plant growth. We found the microbiological genus-level composition of AMF trap-based biofertilizers previously designed for this purpose [19]. Additionally, we examined this biofertilizer’s overall microbial diversity, including the bacterial communities that were not originally intended to be part of it. Furthermore, we delved into the microbial diversity of the biofertilizer plant roots or substrate (sand) micro-niches. Our study highlights the importance of understanding the microbial communities associated with biofertilizers, particularly those with plant-growth promotion capabilities.

## Materials and methods

### Biofertilizer production

The protocol for creating the biofertilizer discussed in this study is detailed in a publication by Trejo-Aguilar and Banuelos (2020) and is available commercially. The biofertilizer development process involves sampling soil from natural ecosystems and searching for AMF spores of multiple species. The original soil of the biofertilizer had a mixture of AMF species, with 4,110 spores per 100 g of soil field before the biofertilizer selection began [19]. According to FAO classification, the original field soil was classified as an Andosol with a sandy texture (27% sand, 22% clay, 22% silt), 7.81% soil organic matter, and pH 5.84 (2:1 soil: water). Additionally, the soil had 0.40 mg kg^-1^ N and 30 mg kg^-1^ P.

The biofertilizer production spans over 16 months, the soil is mixed with sterilized sand, and plants are used as microbial traps. The AMF spores are recovered from the roots and preselected to promote the fast-growing model plants in sterile soil. Quartz sand and pumice are inoculated with host roots and soil. The species used as plant traps were: *Brachiaria brizantha*, *Crotalaria juncea*, *Tagetes erecta*, and *Canavalia ensiformis*.

After two to three months, watering is stopped to induce sporulation, and the roots are separated from the substrate, which is sieved and finely crushed in a mill to get a mixture of substrate and roots. Then the biofertilizer is packaged for commercial use. The resulting mixture has sporulating microbes resistant to desiccation, and the biofertilizer’s activity is tested in fast-growing plants and their controls (Fig 1).

In this study, we collected fresh roots (*Brachiaria brizantha*, *Crotalaria juncea*, and *Canavalia ensiformis*) and substrate samples. For substrate sampling, we used sterile plastic bags. We poured triplicates into filling (50 ml) sterile centrifuge tubes, then fast-frozen them using liquid nitrogen and stored them at -80°C until metagenomic DNA extraction.

### Metagenomic DNA template extraction and amplicon sequencing

We sorted the biofertilizer samples into four groups by their source: fungi from the substrate, fungi from the roots, bacteria from the substrate, and bacteria from the roots. All plant roots and substrates samples were processed as previously described [34–36]. Each sample was extracted in triplicates, using approximately 0.25 g for each. The washing steps of the mechanically broken roots into phosphate-buffer saline solution (PBS) at 7.5 pH were then vigorously mixed and sonicated to get the rhizosphere pellet (1,300 x *g* 10 min). Afterward, and following the manufacturer’s protocol, the metagenomic DNA was extracted using MoBio PowerSoil^®^ DNA Isolation Kit (MoBio Laboratories, Solana Beach, CA, USA). Fungal diversity was assessed by PCR amplification of the ITS region using primers ITS1 (5’-TCGTCGGCAGCGTCAGATGTGTATAAGAGACAG-TCCGTAGGTGAACCTGCGG-3’) and ITS4 (5’-GTCTCGTGGGCTCGGAGATGTGTATAAGAGACAG- TCCTCCGCTTATTGATATGC-3’) indicated by the Illumina’s protocol. The 16S rRNA gene V3-V4 region was amplified by PCR using primers MiSeq341F (5’-TCGTCGGCAGCGTCA GATGTGTATAAGAGACAG-CCTACGGGNGGCWGCAG-3’) and MiSeq805R (5’-GTCTCGTGGGCTCGGAGATGTGTATAAGAGACAG-GACTACHVGGGTATCTAATCC-3’). Both primers for 16S and ITS included 5’ overhangs for sequencing libraries in Illumina. Amplification of ITS region and 16S rRNA V3-V4 region followed the metagenomic DNA denaturation at 94°C for 3 min; then 20 denaturation cycles at 95°C for 30 s and extension at 72°C for 30 s. PCR reactions were done using *Pfx* polymerase (Invitrogen, Thermo Fisher Scientific, Waltham, MA, USA). Triplicate DNA extractions were used for PCR reactions, then mixed and purified with the SV Wizard PCR Purification Kit (Promega, Madison, WI, USA). Sequencing of amplified regions was performed on Illumina^®^ MiSeq^™^ (Illumina, San Diego, CA, USA) with 2 x 300 bp paired-end configuration at the *Laboratorio Nacional de Genómica para la Biodiversidad* (UGA-Langebio) for ITS region, and at the *Unidad de secuenciación Masiva* from *Biotechnology Institute*, *UNAM* for 16S rRNA gene.

### Sequence processing and data analyses

The complete bioinformatics and statistic procedures are available on GitHub (https://github.com/Cristobhal/Biofertilizer), which include modifications to the previously reported protocols used for ITS and 16S rRNA gene analyses [37]. In brief, 16S V3-V4 raw sequences were quality checked and trimmed 250 bp length using FASTX-Toolkit because sequencing quality dropped in both paired-end reads [38]. Sequence assembly was performed with Pandaseq [39] using a quality threshold of 0.95, a minimum length of 250 bp, and a minimum overlap of 15 bp. ITS1-ITS4 amplified sequences were pair-merged using CASPER [40] or Pandaseq [39], considering the assembly of overlapping ITS fragments and merging of forward and reverse reads for non-overlapping ITS, as detailed in [41]. Independent analysis of 16S and ITS sequences was also done using DADA2 [42] from denoising to Amplicon Sequence Variants (ASVs) and taxonomic annotation. Operational taxonomic units (OTUs) were clustered using cd-hit-est at 97% of identity [43], cd-hit is an OTU calling method implemented in suites like QIIME [36] and Amplicon Sequence Variants (ASVs) using DADA2 (v1.14.1) [42]. Chimeras, mitochondrial, and chloroplast sequences were removed. ITS sequences were annotated against the UNITE v9 database [44] using BLAST [45], while 16S rRNA sequences against the SILVA v138 database [46] using a naive Bayesian classifier implemented in DADA2 [42]. Alpha diversity analyses of fungal and bacterial communities were calculated using phyloseq [47] and vegan [48], ggplots2 [49], and statistical analysis in R [50].

## Results

### The biofertilizer exhibited distinct diversity patterns between fungi and bacteria

This descriptive study’s main goal was to inventory the microbial diversity of fungi and bacteria in the biofertilizer. We found two main niches for the microbes: roots and substrate. We opted for deep sequencing of the overall microbial community. Regarding the bacterial 16S rRNA gene amplicons, we got 842,909 sequences from the substrate and 902,266 sequences from the roots. We found 6,971 OTUs in the substrate and 8,819 OTUs in the roots. No differences (*X*^2^, *p =* 0.1573) were seen against expected OTUs richness as the Chao1 was 7,359 ± 29 for substrate and 8,982 ± 16 for roots (Table 1), and we assume a fair coverage of the overall diversity. We found larger Shannon diversity index values for roots (H’ = 7.62) than for the substrate (H’ = 6.08).

**Table 1:** Sequencing outputs and alpha diversity indexes.

	Substrate-associated fungi (ITS)	Roots-associated fungi (ITS)	Substrate-associated bacteria (16S rRNA gene)	Roots-associated bacteria (16S rRNA gene)
Raw paired sequences	62,587	56,566	842,909	902,266
OTUs (97%)	646	397	6,971	8,819
Chao 1 (±SE)	655 ± 4	452 ± 14	7,359± 29	8,982 ± 16
Shannon (H’)	4.7	3.1	6.08	7.62
Pielou’s evenness (J’)	0.728	0.520	0.687	0.838
Simpson	0.97	0.873	0.98	0.99

From a total sequencing of 62,587 fungal ITS reads from substrate and 56,566 from plant roots. We found 646 fungal OTUs in the substrate and 397 in the plant roots. The overall coverage is fair as the observed OTUs did not differ from expected (*X*^2^, *p =* 0.1573) Chao1 estimated richness at 655 ± 4 in the substrate and 452 ± 14 for plant roots (Table 1). The Shannon index showed that the fungal diversity in the substrate (H’ = 4.7) was higher than that in the roots-associated fungi (H’ = 3.1; Table 1).

Opposite to fungal communities, diversity from the roots (H’ = 7.62) was higher than from the substrate (H’ = 6.08). Since Shannon’s diversity index is not a scalar variable, as it expresses richness and evenness, it is suggestive and is not directly comparable between communities. However, when considering evenness as Pielou’s index (J’), it shows larger evenness for root-associated bacteria (J’ = 0.838) than the substrate (J’ = 0.687). With the fungal evenness, it is more prominent in the substrate (J’ = 0.728) than in the roots (J’ = 0.520). These results show that mixing substrate and roots maximizes biofertilizers’ fungal and bacterial diversity. In addition to the described fungal diversity, we found OTUs from plants and microeukaryotes (S1 Fig).

### Microbial compositions of biofertilizer communities

**Fig 2:**
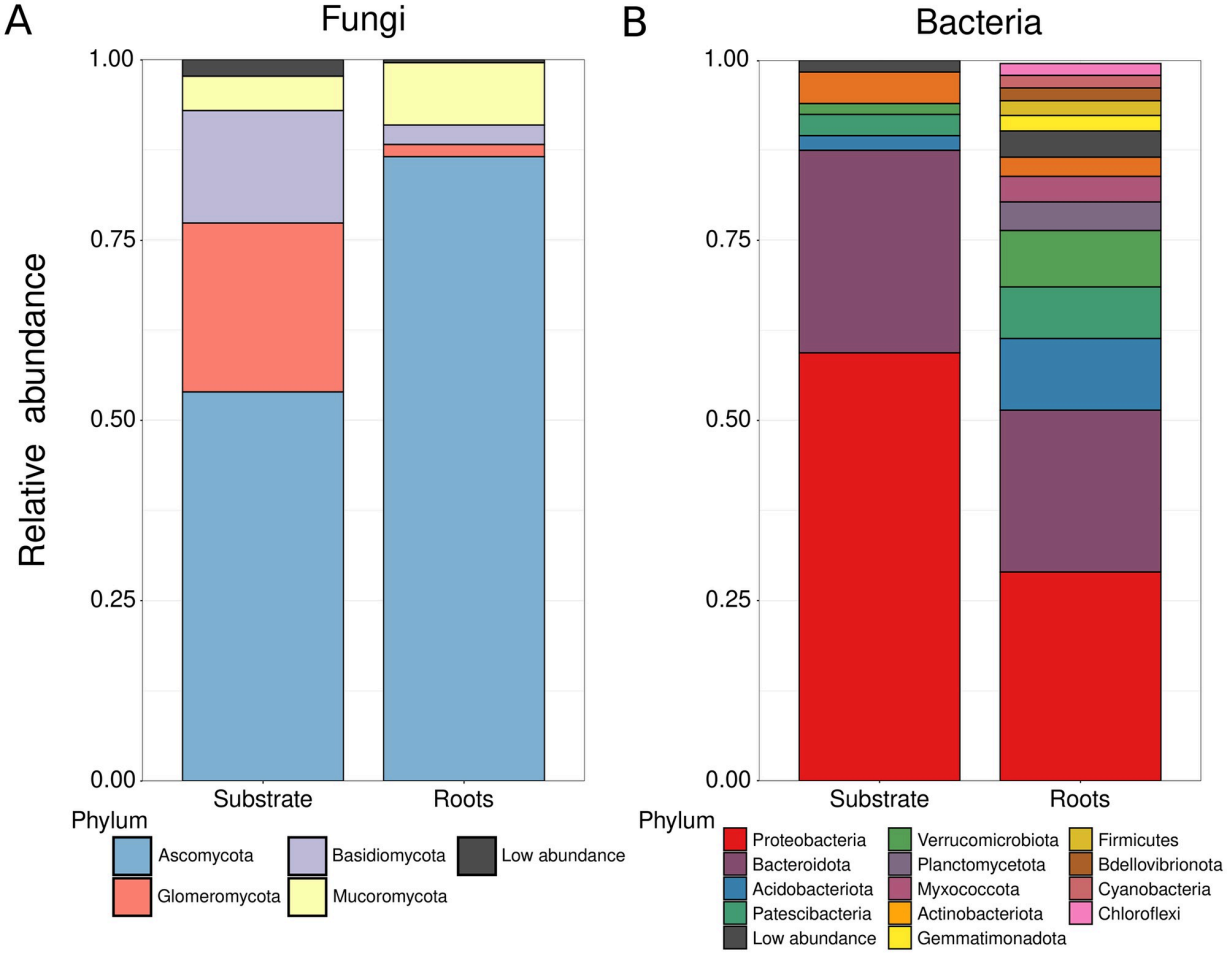
Biofertilizer’s overall diversity in substrates and plant-root association. Phyla diversity of fungal (A) and bacterial communities (B) from the biofertilizer’s substrate or plant roots associated.

The fungal Ascomycota were dominant, followed by Glomeromycota, Basidiomycota, and Mucormycota (Fig 2). However, Ascomycota had a higher relative abundance (RA; RA = 0.54) in the substrate samples than in the roots (RA = 0.86). Glomeromycota and Basidiomycota were more abundant in the substrate (RA = 0.23 and 0.15) than in the roots, where they were drastically reduced (RA = 0.02 and 0.03). Conversely, Mucoromycota was less abundant (RA = 0.05) in the substrate than in the roots (RA = 0.09).

Bacterial communities were dominated by *Proteobacteria*, *Bacteroidota*, *Verrucomicrobia*, *Acidobacteria*, *Patescibacteria*, and *Actinobacteria* (Fig 2B). In the substrate, the phyla *Proteobacteria* (RA = 0.593) and *Bacteroidota* (RA = 0.281) dominated, while *Patescibacteria* (RA = 0.029), *Acidobacteria* (RA = 0.020), and *Actinobacteria* (RA = 0.016) were in low abundance. In the roots, *Proteobacteria* (RA = 0.289) and *Bacteroidota*(RA = 0.224) were reduced compared to the substrate, while Acidobacteria (RA = 0.099) increased. *Verrucomicrobiota* was negligible (RA = 0.015) in the substrate but in a considerable abundance (RA = 0.078) in the substrate (Fig 2).

**Fig 3:**
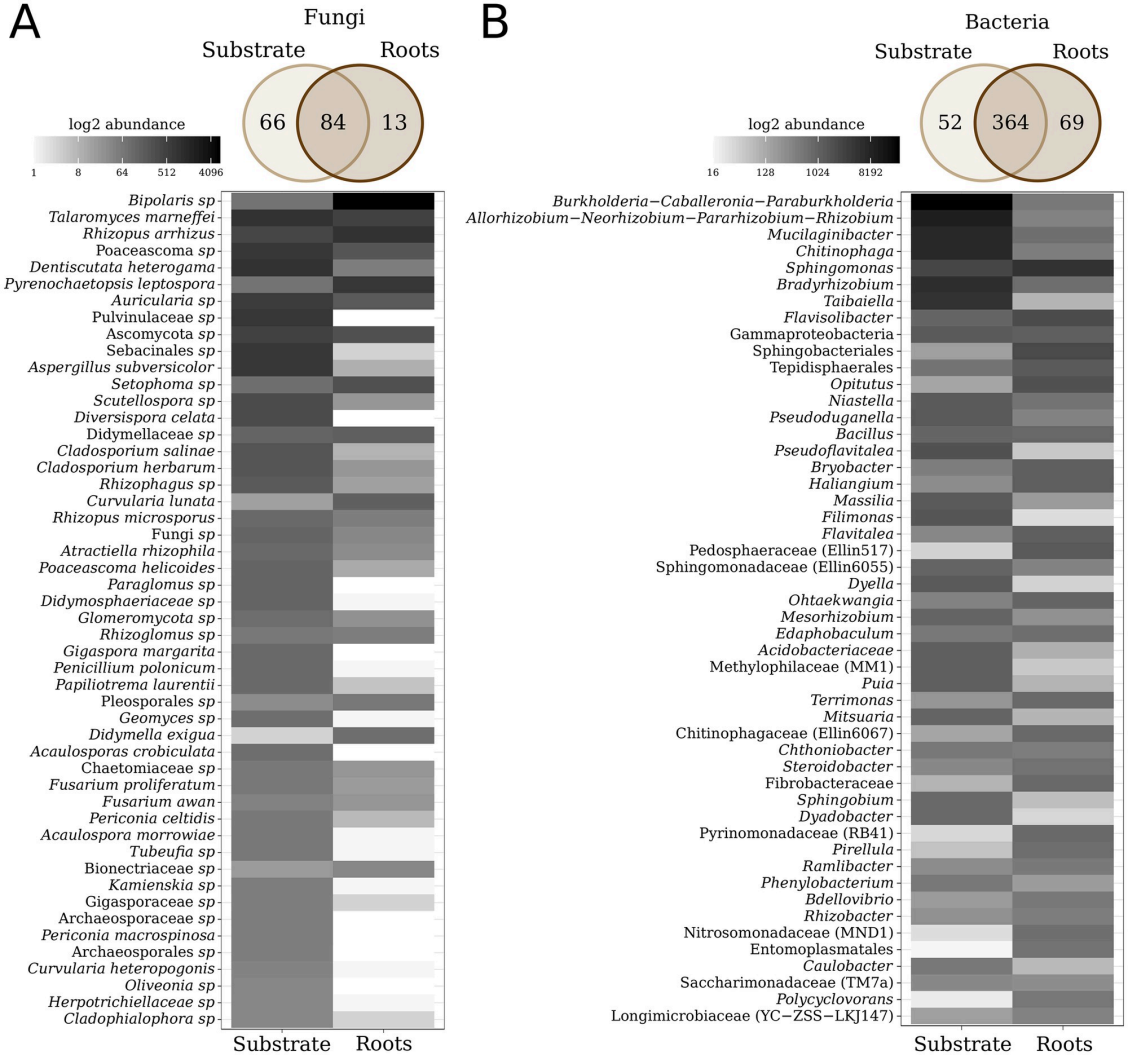
Microbial consortia in biofertilizers. The fungal species (A) and bacterial genera communities (B) present in the biofertilizer are depicted in Venn diagrams, illustrating the number of taxa exclusive to, and shared between, the substrate and the plant roots. Additionally, heat maps display the relative abundance of the most prevalent taxa.

The ITS region sequence analysis allowed us to find 163 fungal species. Of them, 66 were exclusive from the substrate, 13 from the plant roots, and 84 were shared between both samples (Fig 3A, upper section). *Bipolaris*, *Talaromyces marneffei*, *Rhizopus arrhizus*, *Poaceascoma*, *Dentiscutata heterogama*, *Pyrenochaetopsis leptospora*, *Auricularia*, *Aspergillus subversicolor*, *Setophoma*, *Scutellospora*, *Diversispora celata*, *Cladosporium salinae*, *Cladosporium herbarum*, *Rhizophagus*, *Curvularia lunata*, *Rhizopus microspores*, *Atractiella rhizophila*, *Poaceascoma helicoides*, *Paraglomus*, and *Gigaspora margarita* were the more abundant fungal species (RA > 0.01) in the substrate or the roots (Fig 3; S1 Table). Remarkably, the overall pattern of the most abundant phyla and fungal species showed similarity when analyzing the samples, considering only the forward sequences, concatenating the merged and unmerged sequences, and even using ASVs (S2 Fig).

The 16S rRNA gene analysis revealed 485 bacterial genera, of which 52 were exclusive from the substrate, 69 were exclusive from the root, and 364 were found in both (Fig 3B, upper section). The main bacteria groups were *Parabulkholderia-Caballeronia-Burkholderia*, *Allorhizobium−Neorhizobium−Pararhizobium*−*Rhizobium*, *Mucilaginibacter*, *Chitinophaga*, *Sphingomonas*, *Bradyrhizobium*, *Taibaiella*, *Flavosolibacter*, *Opitutus*, *Niastella*, *Pseudoduganella*, *Bacillus*, *Pseudoflavitela*, *Bryobacter*, *Haliangium*, *Massilia*, *Filimonas*, *Flavitelea*, *Dyella*, and *Ohtaekwangia*were dominant in the substrate or the roots (RA > 0.01; Fig 3; S1 Table). The complete lists of taxa from fungal and bacterial communities are available in S1 Table. Our OTUs and ASVs results were consistent with the most abundant bacterial genera. Therefore, our methods were suitable for describing the inventory of microorganisms in the biofertilizer (S3 Fig).

## Discussion

### Assembly of microbial communities

This work evaluated the diversity levels of the biofertilizer microbial communities, showing that its production optimizes the fungal and bacterial communities having plant growth-promoting microorganisms. While bacterial diversity was increased between the substrate and the roots, fungal diversity followed an inverse pattern (Table 1). The increase in diversity in root bacterial communities (Table 1) may be attributed to the nutrient-rich environment established in the rhizosphere by the plant metabolite secretion [51]. Earlier studies have shown that rhizospheres host higher bacterial alpha diversity than soils [35, 52].

A common garden experiment found that the microbiomes of ruderal plants show higher alpha diversity than their soils. Similarly, when the same soils are tested on domesticated plants, the alpha diversity decreases in the rhizosphere, suggesting that the rhizosphere supplies a microenvironment that supports bacterial diversity [35]. Our findings suggested that the biofertilizer’s sandy substrate served as a source of bacterial inoculum and enriched the biodiversity through the presence of plant roots, which acted as a system for attracting and promoting bacterial growth. Notably, specific genera such as *Sphingomonas*, *Flavisolibacter*, and *Opitutus* were more abundant in the roots than in the substrate. Since the host plants *C*. *juncea* and *C*. *ensiformis* are legumes, the root-exudated flavonoids could attract exclusive rhizobia from the roots to induce nodulation [53]. Some bacteria, such as *Sphingomonas*, are also vertically inherited in several generations of the plant *Crotalaria pumila* in the seed microbiome [54].

The reduction of root fungal diversity may be explained by the selection process driven by the roots. In which inoculated fungi are selected by their affinity with root-released metabolites and host genotype [51, 55]. We found 163 fungal species in the inoculated substrate. However, only 13 were detected in the roots (Fig 3A), like the previously observed reduction of the fungal diversity between the soil and the root found by other works [56, 57]. Although plants like *Brachiaria*, *Crotalaria*, and *Canavalia* are known for setting up arbuscular mycorrhizal interactions that improve their growth under unfavorable conditions [58–61], we did not detect *D*. *celata* and *G*. *margarita* in the roots (Fig 3A; S1 Table; Table 2). The low abundance of Glomeromycota associated with roots is a common pattern found in several cultivated species, such as *Agave* [62], sugarcane [54], cactus [55], and wheat [58, 62–64]. Nonetheless, the substrate kept a high proportion of AMF compared with soils [65, 66], reflecting a thriving selection of fungal symbionts other plant species can recruit.

**Table 2:** Fungi with a role in plant growth promotion in the biofertilizer.

Species (based on ITS best match)	Relative abundance in the substrate	Relative abundance in roots	Role in plant growth promotion	References
*Dentiscutata heterogama*	*0*.*077*	*0*.*005*	Mycorrhizal fungi; Protection against nematodes; improves nutrient uptake (N, P, K, Zn, and Fe) in a mycorrhizal consortium.	[82]
*Rhizopus arrhizus*	*0*.*038*	*0*.*082*	Phosphorus mineralization by phytase; chitosan production.	[83, 84]
*Diversispora celata*	*0*.*029*	*0*	Mycorrhizal fungi; improve phosphorus and nitrogen uptake.	[85, 86]
*Cladosporium salinae*	*0*.*021*	*8x10* ^*-4*^	*Cladosporium sp*. secretes volatile compounds, hydrolytic enzymes, and IAA; solubilizes phosphorus and zinc; and antagonizes pathogens.	[87]
*Atractiella rhizophila*	*0*.*01*	*0*.*003*	Increases height and photosynthetic rate; unknown mechanisms.	[88]
*Gigaspora margarita*	*0*.*01*	*0*	Mycorrhizal fungi; protection against pathogenic fungi; phosphate solubilization.	[89, 90]
*Acaulospora scrobiculata*	*0*.*008*	*0*	Mycorrhizal fungi; increase photosynthetic pigments, leaf N content, and photosynthetic rate.	[91]
*Bipolaris*	*0*.*007*	*0*.*6*	Indole-acetic acid, abscisic acid, gibberellin, and siderophores production; phosphate solubilization.	[92]
*Fusarium proliferatum*	*0*.*006*	*0*.*001*	Indole-acetic acid and gibberellin production.	[93]
*Rhizoglomus sp*.	*0*.*005*	*0*.*005*	Mycorrhizal fungi; unknown.	[94]
*Periconia macrospinosa*	*0*.*004*	*0*	Improve nitrogen uptake.	[95]
*Aspergillus flavus*	*0*.*002*	*1*.*8x10*^*-4*^	Phosphate solubilization.	[96]
*Papiliotrema laurentii*	*0*.*001*	*4*.*5x10*^*-4*^	Phosphate solubilization; Increase N and P when inoculated with mycorrhizal fungi; stimulates root growth; improves nodule size and nitrogen fixation.	[73, 75, 97]
*Glomus sp*.	*0*.*001*	*0*	Mycorrhizal fungi; phosphate solubilization; tolerance to salt stress; resistance against pathogens.	[98, 99]
*Didymella exigua*	*2*.*6x10*^*-4*^	*0*.*0008*	Protection against pathogenic fungi.	[100]
*Rhizophagus custos*	*1*.*7x10*^*-5*^	*9x10* ^*-5*^	Mycorrhizal fungi; unknown	[101]
*Exophiala sp*.	*8*.*7x10*^*-5*^	*1*.*8x10*^*-4*^	Promotes growth in cadmium-contaminated soils; enhances phosphorus absorption.	[102]

*⁠*Fungi are sorted according to their relative abundance in the substrate.

We must highlight that we divided the samples of substrate and roots to understand plant-microbe interactions in line with other works [57, 67] as a process of microbial colonization from soil to roots [55]. Despite the bioinoculant being composed of dried roots and substrate, particles of the substrate can adhere to them during processing and play a role in the observed positive effects on plant phenotype. Therefore, a microbial characterization of the biofertilizer should consider the beneficial microbes in both the substrate and the roots.

**Fig 4:**
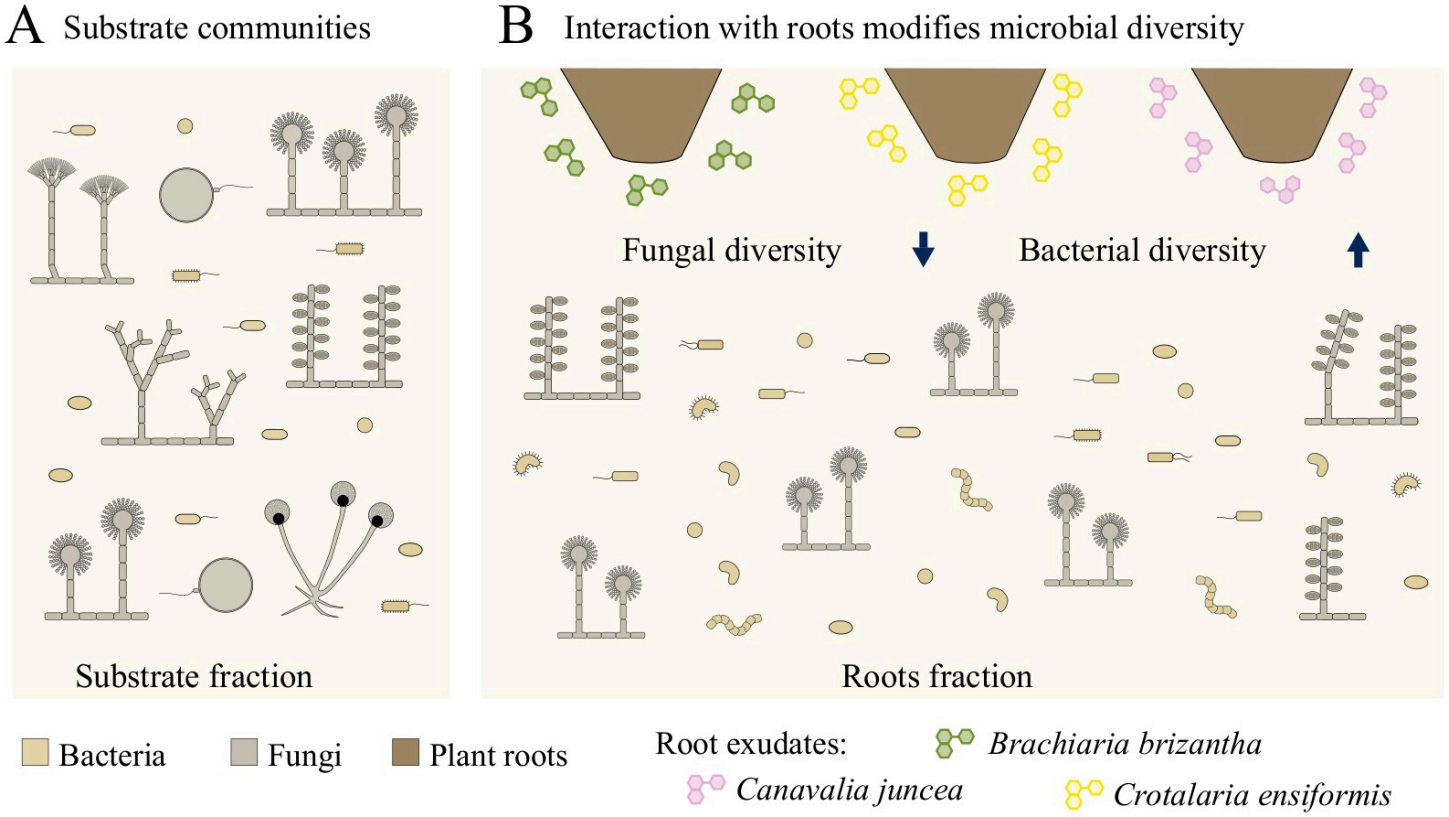
A comprehensive model explains the biofertilizer’s observed bacterial and fungal diversity. (A) Initially developed for fungi and bacteria, the consortia formation process involves introducing bacteria to the substrate through the first mycorrhizal fungi inoculum. (B) Subsequently, the plants actively select their fungal partners, reducing fungal diversity near the roots. Interestingly, the root-associated bacteria displayed greater diversity, likely due to the nutrient-rich environment created by the plant root exudates.

We proposed a model that explains the assembly of bacterial and fungal communities in the biofertilizer (Fig 4). The inoculation of mycorrhizal fungi added bacteria to the sandy substrate. During the plant growth process in greenhouses, beneficial microorganisms are intentionally added to biofertilizers, and some microbes are inadvertently introduced through natural means. For instance, other unintended microbial inoculations can occur through watering, which often uses non-sterile water, or environmental aerosols and dust. As the plants are planted to the substrate, they filter the fungi based on their ability to interact with *B*. *brizantha*, *C*. *juncea*, and *C*. *ensiformis*, reducing fungal diversity in the root-associated communities. However, plants exude metabolites to the substrate, creating a nutrient-rich niche that boosts bacterial diversity [51, 68]. Although the root-associated communities have bacteria and fungi, our results suggest that incorporating the substrate fraction into the biofertilizer is relevant to supporting the whole diversity of the original fungal community. Fungal diversity from the substrate includes arbuscular endomycorrhizal fungi with agricultural relevance from the phylum Glomeromycota [69].

For this reason, both the substrate and the roots are essential to the biofertilizer since their synergistic effect provides a broad spectrum of microbes that can promote plant growth in several plant species. In addition, the biofertilizer process resembles host-mediated microbiome engineering (HMME). HMME is a multigenerational process that focuses on the host and sub-selects beneficial microbes at a community level rather than individually. Since the HMME can be targeted to increase plant tolerance to environmental stress [70], new biofertilizers could be designed by trap cultures and the positive selection of stress-resistant phenotypes.

### Microbes with reported plant growth promotion activity

According to the literature, ITS and 16S rRNA gene sequencing allowed us to find taxa that may handle plant growth promotion (Tables 2 and 3). Arbuscular mycorrhizal species such as *Dentiscutata heterogama*, *Diversispora celata*, and G. *margarita* were found in the substrate (Fig 3; Table 2; S1 Table). Besides the direct benefits to plants, mycorrhizal fungi contribute to setting up other plant growth-promoting microorganisms [71, 72] found in higher abundance (Fig 3; Tables 2 and 3; S1 Table). *Papiliotrema laurentii* can interact with *Funneliformis mossae* to enhance nutrient content in roots and leaves [73] or with other mycorrhizal species to increase nodule size and nitrogen fixation [74].

**Table 3:** Bacteria with a role in plant growth promotion in the biofertilizer.

Genus	Relative abundance in the substrate	Relative abundance in roots	Role in plant growth promotion	References
*Burkholderia*	0.218	0.006	Nitrogen fixation, phosphate solubilization; indole acetic acid production; antifungal activity.	[103]
*Rhizobium*	0.081	0.004	Nitrogen fixation, phosphate solubilization, indole acetic acid, gibberellin production, and induced systemic resistance.	[104–106]
*Bradyrhizobium*	0.047	0.008	Nitrogen fixation; indole acetic acid production; phosphate solubilization.	[104, 107]
*Sphingomonas*	0.020	0.050	Indole acetic acid and gibberellin production.	[108]
*Massilia*	0.011	0.002	Indole acetic acid and siderophore production.	[109]
*Dyella*	0.011	4.6x10^-4^	Phosphate solubilization	[110]
*Mesorhizobium*	0.009	0.003	Nitrogen fixation; phosphate and potassium solubilization; Indole acetic acid and siderophores production;	[111]
*Bacillus*	0.008	0.01	Nitrogen fixation, phosphate solubilization, induction of iron acquisition genes from plants; alteration of plant growth hormone homeostasis; drought and salt stress resistance; production of antimicrobial compounds; induced systemic resistance.	[112]
*Sphingobium*	0.007	7.8x10^-4^	Improve tolerance to cadmium	[113]
*Dyadobacter*	0.007	4.2x10^-4^	Nitrogen fixation; phosphate solubilization	[114, 115]
*Chitinophaga*	0.006	0.005	Phosphate solubilization; production of chitinase against pathogenic fungi.	[116]

*⁠*Bacteria are sorted according to their relative abundance in the roots.

Other highly abundant fungal species are *Talaromyces marneffei* and *Aspergillus subversicolor* (Fig 3 and S1 Table). To our knowledge, *T*. *marneffei*, previously classified as *Penicillium marneffei*, is a human opportunistic pathogen in immunocompromised patients [75] with no records in plant microbiomes. Species-level identification based solely on the ITS region may need to be revised. While the observed OTUs belong to the *Talaromyces* genus, further evidence is needed to confirm their specific species. Additionally, figuring out their pathogenicity requires consideration of the host’s physiology. However, some *Talaromyces* species enhance plant growth by controlling pathogens [69, 76–78] or producing antioxidant enzymes and osmolytes [79]. Although *A*. *subversicolor* was isolated from coffee [80], there is little information about the species. On the other hand, some species of *Aspergillus* can help agricultural production due to their ability to solubilize and mineralize phosphorus and produce secondary metabolites and phytohormones [81].

We suggested that biofertilizer improves plant growth through four main mechanisms: nutrient uptake, phytohormone production, stress tolerance, and resistance to pathogens (Tables 2 and 3). The primary mechanism for plant growth promotion by fungus seems related to phosphorus acquisition mediated by phosphate solubilization [90] and phosphate mineralization [83]. Although we found some phosphate-solubilizing bacteria [106], our dataset suggests that while fungi were involved in phosphorus nutrition, bacteria may play a key role in nitrogen uptake by nitrogen fixation [107, 111, 117]. Both fungi and bacteria can produce siderophores for iron uptake [109, 111, 118]. Regarding stress response, there are reports of microorganisms, such as *Glomus* and *Bacillus*, involved in salt and drought stress tolerance [99, 108]. *Bacillus subtilis* increases the tolerance of plants to salt and drought stress by modulation of abscisic acid, one of the main phytohormones for stress response [112]. We found several microorganisms involved in resistance against pathogens. For instance, some *Cladosporium* isolates showed antagonist activity against plant pathogens such as *Rhizoctonia solani*, *Fusarium graminearum*, *Sclerotinia sclerotiorum*, and *Botrytis allii*. Additionally, they secrete hydrolytic enzymes that may act against the fungal cell walls of these pathogens [87].

Finally, some taxa we considered plant growth promoters have species with pathogenic activity. For example, some *Bipolaris* and *Fusarium* species handle diseases that can cause rot in plant organs [119, 120]. However, we found that some species of *Bipolaris sp*. *CSL-1* produces indole acetic acid and gibberellins, increasing seedling biomass and chlorophyll content [92]. Some *Fusarium* species could promote growth by phosphate solubilization, synthesis of phytohormones, and siderophore production [121, 122]. Alternatively, several biofertilizer microbial communities could produce metabolites to antagonize pathogens from the same community [99, 112, 123]. Earlier works suggest that antagonistic interactions between bacteria and fungi may promote plant growth [124], and resource competition between closely related species (non-pathogenic vs. pathogenic) may also exclude pathogens from plant roots [125]. In addition, PGPB consortia are composed of mutualistic organisms and have microbes without directly helping plants that play essential roles in their communities [126].

## Conclusions

We investigated the fungal and bacterial diversity of a commercial biofertilizer, which consists of plant roots added to a mycorrhizal-inoculated substrate. Our findings reveal the identification of 182 fungal species and 964 bacterial genera. The dominant fungi were *Bipolaris*, *Rhizopus*, and *Scutellospora*, while the dominant bacteria were *Burkholderia*, *Rhizobium*, *Sphingomonas*, and *Chitinophaga*. Interestingly, these microbes are known to promote plant growth. We have seen that fungal diversity was higher in the substrate, while bacterial communities showed greater diversity in the roots. Our results suggest that initial inoculation supplies a high fungal diversity, while plant incorporation diversifies bacterial communities, giving rise to a wide array of microorganisms that promote plant growth. Moreover, the long-term selection of beneficial microbial communities interacting with plant roots and enhancing their phenotype can lead to the development of new biofertilizers. These biofertilizers could be tailored to address specific issues such as biotic and abiotic stress.

## Supporting information

S1 Fig Eukaryotic diversity. A) Phyla diversity and B) Relative abundance of the non-fungal genus.
(TIF)

S2 Fig Comparative analysis of methods used to evaluate fungal diversity: OTUs, OTUs using only forward sequences, OTUs using concatenation of merged and unmerged sequences, and ASVs. A) Predominant Phyla, B) shared genera, and C) most abundant genera.
(TIF)

S3 Fig Comparative analysis of methods used to evaluate bacterial diversity: OTUs and ASVs. A) Predominant Phyla, B) shared genera, and C) most abundant genera.
(TIF)

S1 Table List of fungal species and bacterial genera. Species and genera are sorted according to their relative abundance in the substrate.
(XLSX)
