# Supplementary figures and images for: A study of microbial diversity in a biofertilizer consortium
Source: PLoS One. 2023 Aug 24;18(8):e0286285. doi: 10.1371/journal.pone.0286285 (PMC10449135; doi:10.1371/journal.pone.0286285)

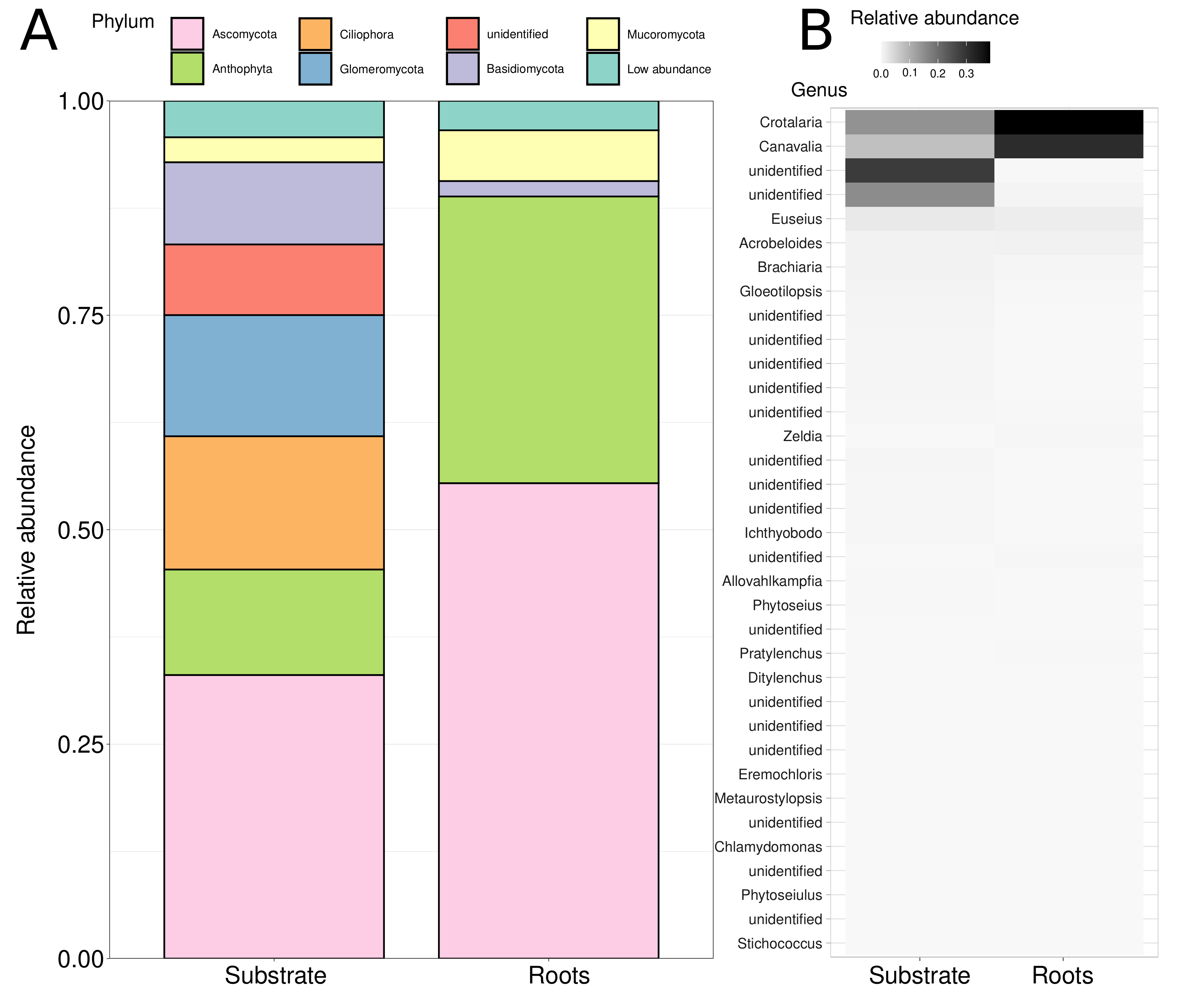

Supplement: S1 Fig — A) Phyla diversity and B) Relative abundance of the non-fungal genus. (TIF) [file pone.0286285.s001.tif]

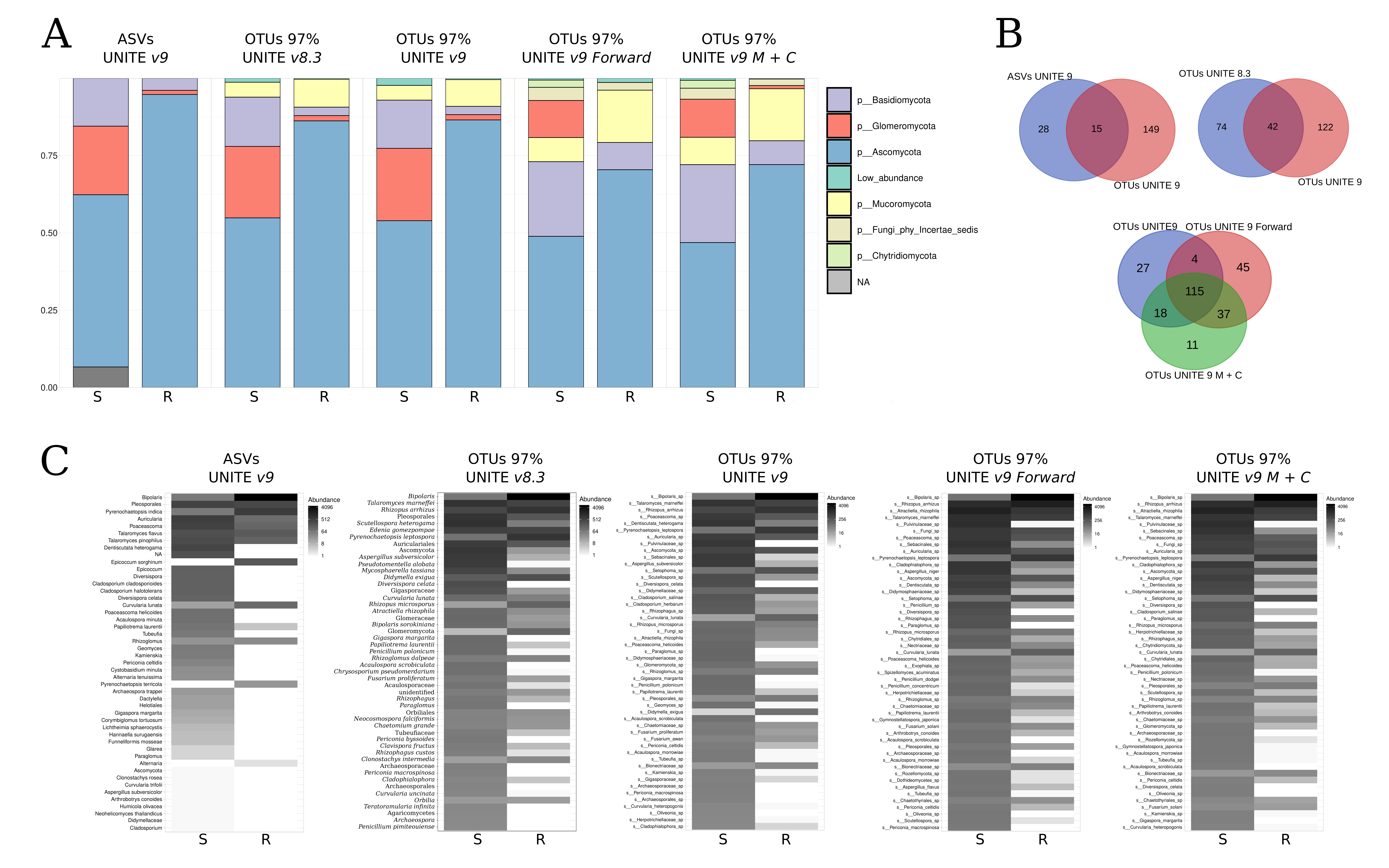

Supplement: S2 Fig — A) Predominant Phyla, B) shared genera, and C) most abundant genera. (TIF) [file pone.0286285.s002.tif]

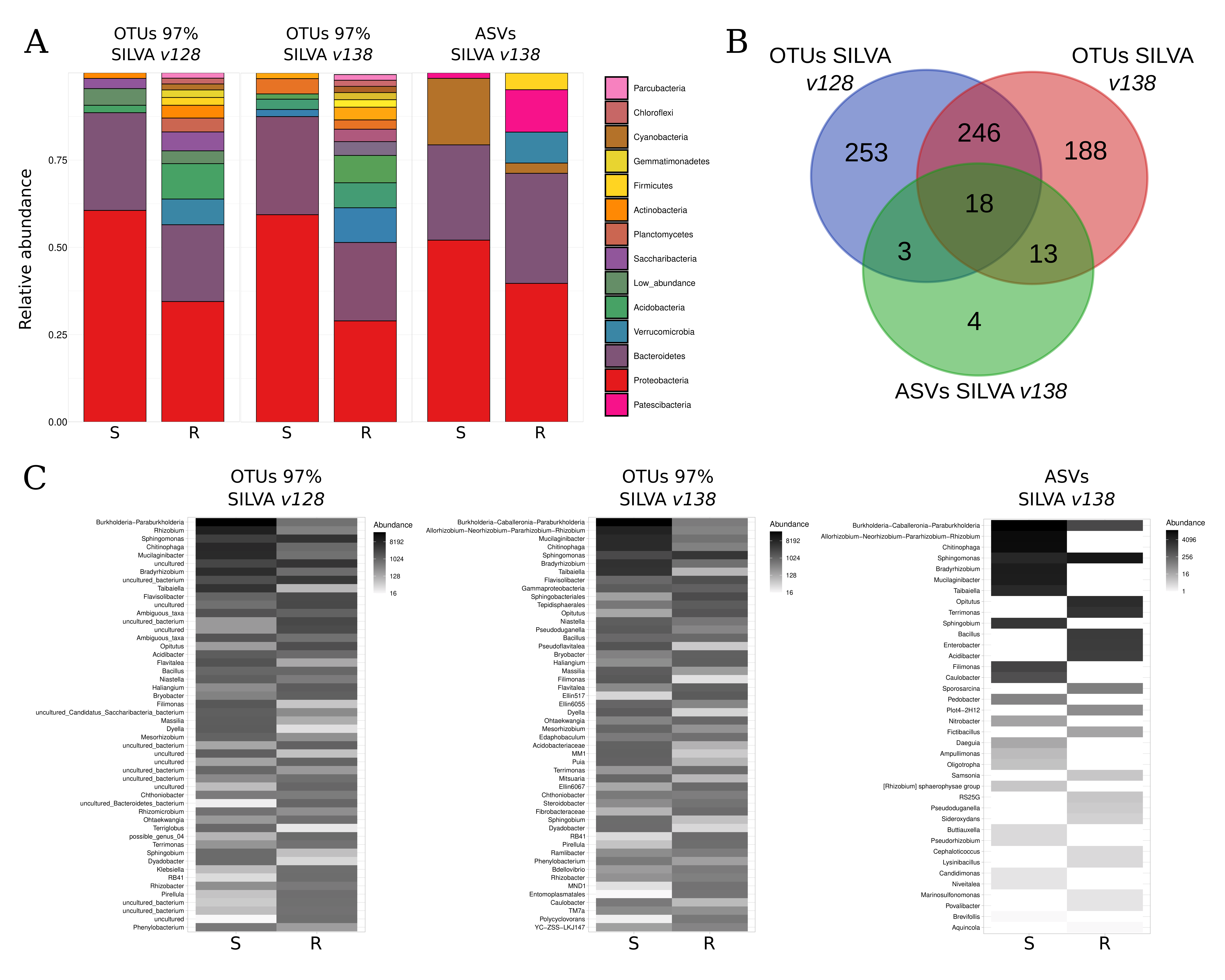

Supplement: S3 Fig — A) Predominant Phyla, B) shared genera, and C) most abundant genera. (TIF) [file pone.0286285.s003.tif]
